# The Manila Declaration on Migration and Health: commentary of the Association of Pacific Rim Universities Global Health Program

**DOI:** 10.1186/s12992-020-0544-0

**Published:** 2020-01-22

**Authors:** Mellissa Withers, Heather Wipfli, Marc Schenker, Tasfia Jahangir, Teodoro Herbosa, Jorge Tigno

**Affiliations:** 1 University of Southern California Keck School of Medicine, Institute on Inequalities in Global Health, 2001 N Soto Street SSB 318G, Los Angeles, CA 90089 USA; 2 University of Southern California Keck School of Medicine, Los Angeles, USA; 3 University of California, Davis, USA; 4 University of Southern California, Los Angeles, USA; 5 University of the Philippines, Manila, Philippines

**Keywords:** Migration, Migrant, Public health, Human rights, Asia-Pacific, Global health, Immigrant

## Abstract

**Background:**

Migration has played, and continues to play, an important role in shaping our global economy. As of 2017, there were 258 million international migrants worldwide, over 100 million of whom came from the Asia-Pacific region. Migration is increasingly recognized as a social determinant of health, as migrants often experience vulnerabilities that make them susceptible to a range of negative health outcomes. Addressing the health and human rights concerns of migrants requires concerted and global efforts from many stakeholders, including universities.

**Methods:**

The Global Health Program of the Association of Pacific Rim Universities (APRU), a non-profit network of more than 50 universities in the region, is an example of an avenue to foster research, innovation, collaborative engagement, and large-scale advocacy around migration and health. In 2017, a special half-day workshop was held in Manila, convening 167 participants from 10 economies and 21 disciplines. The goal of the workshop was to delineate the role of universities in promoting migrant health and well-being. The global health experts from a diverse set of backgrounds collaboratively developed a policy statement to be used to better address migrant health and human rights. The objective of this paper is to disseminate the policy statement, highlighted specific action items that universities can take to protect and promote migrant health.

**Results:**

The Manila Declaration on Migration and Health highlights that universities must ensure that their campuses are safe, supportive, and empowering environments for all migrants and their families. Universities are also urged to capitalize on their educational and research expertise to generate data on migrant experiences and communicate this research to policymakers.

**Conclusions:**

This commentary highlights how institutions of higher education can serve as powerful avenues for promoting migrant health and human rights. Universities can play a vital role in building awareness and sensitivity to migrant challenges and needs, as well as helping to develop policy frameworks appropriate to their diverse contexts to guide, promote, and reinforce commitment to migrant rights and health. Universities should also ensure that their campuses are safe, supportive, and empowering environments for all migrants and their families.

## Background

International migration is a global phenomenon that is growing in scope, complexity, and impact [1]. The United Nations defines an international migrant as someone who has resided outside of his or her country of birth for at least 12 months [2]. As of 2017, there were an estimated 258 million international migrants worldwide, representing about 3.4% of the world’s population. Of these, 150 million were migrant workers and over 100 million were born in Asia [3]. This figure also includes about 4.6 million international students, up from two million in 2000 [4]. It does not include the approximately 740 million *internal* migrants worldwide, most of whom live in the Asia-Pacific region, who experience many of the same adverse health outcomes as migrants who live abroad [5].

The Asian region is an important source of migration, as it is currently home to the most international migrants worldwide (80 million). It also is the greatest *source* of international migrants; 110 million of the estimated 258 million international migrants were born in Asia [3]. Between 2000 and 2017, Asia added more international migrants than any other region, gaining some 30 million international migrants during this period [3]. Within the region, Thailand has the most immigrants (3.6 million), which represent 5.2% of the population. Malaysia, with 2.7 million immigrants, is second, where they represent 8.5% of the population [6]. Across the Pacific Rim, China, India, Mexico, and the Philippines are the top economies of origin for migrants, while the United States, Japan, South Korea, Taiwan, and Singapore, are the major destination economies. Many Asian migrant workers can also be found beyond the Pacific Rim region with significant numbers employed in the Middle East especially in Saudi Arabia, Qatar, and the United Arab Emirates [2, 3].

The rise in global mobility and its positive and negative impact on nations, migrants, families and communities have contributed to the increased attention toward the topic of international migration within the global community. Moreover, the scale and significance of migration throughout the Asian region makes it a critical topic for research, education, and policy. Recognizing the potential role of universities in supporting and advancing this work, the Global Health Program of the Association of Pacific Rim Universities chose to focus on the topic of migration at their 2017 annual conference in Manila, the Philippines. In this paper we describe the key issues discussed, present the final text of the Manila Declaration on the Migration and Health, adopted following the meetings’ debate, and discuss the implications and recommendations about the future role of universities in the area of migration.

### Background

#### The Association of Pacific Rim Universities

The Association of Pacific Rim Universities (APRU) is a non-profit network of more than 50 leading research universities in the region, representing 17 economies in the region. Launched in 2007, the APRU Global Health Program (GHP) includes approximately 2000 faculty, students, and researchers who are actively engaged in global health work. The main objective of the GHP is to advance global health research, education and training in the Pacific Rim, as APRU member institutions respond to global and regional health challenges. Each year, about 300 APRU GHP members gather at the annual global health conference, which is hosted by a rotating member university. The APRU network of university members together represent more than 360,000 employees and more than two million students, many of whom are migrants from throughout the Asia-Pacific. As such, the network recognized that it could potentially play a key role in the advancement of migrant health and rights through effective research, education, and service.

In October 2017, at the annual APRU conference hosted by the University of the Philippines in Manila, a half-day special workshop was held at the conference focusing specifically on human migration and health. The special workshop had three objectives: 1. To give an overview on the topic of migration and health in the region; 2. To delineate the role of universities in protecting and promoting migrant health and rights; And 3. To collaboratively develop a policy statement describing approaches to address this issue to be disseminated to all the participants and key policymakers both in the Philippines, as well as well as globally.

Participants of the workshop included 167 university professors, students, university administrators, government officials, and employees of non-governmental organizations (NGO), from 21 disciplines, including anthropology, Asian studies, communication, dentistry, development, education, environmental health, ethics, international relations, law, library and information science, medicine, nutrition, nursing, occupational health, pharmaceutical science, physical therapy, political science, psychology, public health, and women’s studies. The participants came from 10 economies: Australia, China, Hong Kong, Indonesia, Japan, Mexico, Nepal, the Philippines, Thailand, and the US. The workshop began with presentations on migration and health by researchers from Thailand, the Philippines, and the United States. These presentations and background discussion focused on the history of migration in the region and the health and human rights concerns of migrants.

#### History of migration in the Asia-Pacific

Asian migration is not new; it dates back to the colonial period when indentured workers were mostly recruited by force. In many places around the world, such as some economies in Southeast Asia, Chinese workers played an important intermediary role during colonialism as trading minorities, which led to the development of ethnic networks, which in turn spurred more migration in these economies as family members joined the migrants in their new economies in both the Global North and South [7]. Thus, the nineteenth century was marked by the migration of thousands of people from China and Japan to the United States, Canada, and Australia [7]. The growing number of migrants sparked anti-immigrant sentiments, which led to the passing of restrictive migration policies in many host economies in the early twentieth century [7]. However, Asians continued to migrate, often as a result of political struggles in their own economies. Forced internal displacement also became a major problem in Asia as a result of urbanization and development projects (e.g. large dams), environmental degradation, and natural disasters (e.g., volcanoes and floods). Vulnerable groups, such as indigenous populations or ethnic minorities, also experienced displacement because of sociopolitical challenges during that period [7].

In the 1950s and 1960s, many anti-immigration policies were rescinded, leading to an influx of foreign investment and trading networks in the region. War and conflict in Asian economies like Korea and Vietnam led to large-scale refugee movements, as well as migration of brides of servicemen and their family members through family reunification policies [6]. During the 1980s and 1990s, globalization and increased demand for labor in the emerging industrial economies of Asia led to the exponential growth of labor migration, including skilled workers [7]. Migration of female domestic workers to economies like Malaysia, Singapore and Hong Kong also surged. This demand was met by women largely from the Philippines, Indonesia and Bangladesh [7]. Since the 1990s, marriage migration across Asia has also increased [7]. Today, women make up nearly one-half of the total migrant population from Asia [3].

The twenty-first century has seen rapidly increasing migration and population diversity globally. At the same time, the increasingly negative social and political discourse about migrants has once again given rise to anti-migrant sentiment and restrictive policies in many economies around the globe [4, 8]. Economies that have high numbers of their citizens working overseas are grappling with how to better protect them. As migrants will continue to play an important role in shaping the region and the world in the twenty-first century, a concerted global effort is therefore imperative to protect and promote their health and human rights, regardless of documentation status or reasons for migrating [6–8]. Universities can play an important role in fostering dialogue, research, awareness, and advocacy towards this goal.

#### The health and human rights concerns of migrants

The major drivers of migration include income inequality, conflict, and climate change [4]. The majority of migrants leave their home economies in search of a better job or education. For those without resources and support, migration may be the only available to escape poverty and instability. Other migrants and refugees are forced to flee persecution, violence, or human rights violations, such as torture and discrimination based on ethnicity, sexual orientation, or other minority status [8, 9]. These journeys, which begin with the hope for a better future, often also warrant fear and danger. It was estimated in 2010 that there were 50 million irregular (or informal) migrants worldwide, meaning that they do not have legal protection from the host economies [3]. This exposes them to even greater risks and vulnerabilities. For example, it is estimated that about 62% of the global population in modern day slavery, about 20 million people total, are in the Asian region, working in industries like agriculture, fishing, and commercial sex [10].

While many documented migrants are formally hired as skilled workers and professionals, the vast majority of migrant workers are employed in low-skilled, low-paying, and low-status jobs. Increasingly, migrants are performing the dirty, dangerous, and difficult jobs that local workers refuse to do. Although these undocumented workers may help meet the demand for labor in their host economies, they often experience negative mental, social, and physical consequences as a result. Their positions as foreigners with limited power and resources often places them in precarious, unstable positions where they can be exploited [8, 11]. Undocumented immigration status, language barriers, social exclusion, and lack of migrant-inclusive initiatives and policies contribute to major health disparities for migrant populations [10, 11]. Increasingly, migration is gaining attention as a social determinant of physical health, mental health and social well-being. Policies that can address migration-related health vulnerabilities and provide better access to health care services are requisites for achieving the Sustainable Development Goals [11]. In addition, migration can also bring significant improvements in physical, mental and social well-being, which can enable them to make significant social and economic contributions in the host economies, as well as back home [11]. For example, global remittances to low- and middle-income economies in 2017 totaled $466 billion; the top remittance receiving economies were India, China, and the Philippines [4]. The International Office on Migration reports that “delivering equitable access for migrants will reduce health and social costs, improve social cohesion and, most importantly, will contribute to healthier migrants in healthier communities.” [9] As the United Nations Secretary-General Antonio Guterres recently pointed out, “migration powers economic growth, reduces inequalities and connects diverse societies.” [12]

## Methods

Following the presentations of background information, small groups of six to eight people were formed to draft a policy statement over about one hour, focusing on the role of universities in promoting the health and well-being of migrants. Groups were instructed to take notes and a one-hour debriefing session with the entire group took place after the small group work. The notes from the small group discussions and the debriefing were then compiled and reviewed by the co-authors of this paper in order to draft the statement. The draft was then presented at a plenary session of the conference two days later and was also circulated by email to all workshop participants for comments and edits over about three months. All comments were reviewed and incorporated into the final version, which was collaboratively written by all co-authors over the next six months.

## Results

The final “Manila Declaration on the Migration and Health” was approved by the workshop participants. The final text reads:

We, the participants of this workshop:

- Acknowledge that migration is an inevitable and normal global phenomenon
- Appreciate the mutual benefit that can be gained in both sending and receiving economies and the significant positive contribution that migrants can make to society
- Recognize the significant challenges faced by migrants as well as legitimate security concerns in host communities
- Recognize migration as a social determinant of health
- Affirm the human rights of all migrants around the world
- Acknowledge that migrants in general have poorer health outcomes than native-born
- Acknowledge the substantial existing evidence and international agreements in place

We commit to:

- Advocate for a more concerted and coordinated global effort to protect and promote the health and human rights of migrants
- Develop clear, consistent, and sustainable policy frameworks appropriate to our diverse contexts that guide, promote, and reinforce our commitment to migrant rights and health, including providing health services, legal support and educational programs to migrant employees and students and their families
- Support the development of curricula and new courses and provide trainings across disciplines and sectors to improve capacity and increase cultural competency within our university communities and among those serving migrants
- Conduct holistic, participatory research and generate data on migrant experiences and health status including evidence on gender and mental health and ensure that this research is communicated to policymakers
- Provide educational and employment opportunities to migrants and their families
- Partner with the media and community-based organizations to build awareness and sensitivity to migrant challenges and needs in our diverse communities through the dissemination of creative resources including case studies, digital storytelling, and social media posts
- Promote our shared institutional values of equity and diversity by ensuring that we provide safe and supportive workplace to all employees and that our campuses are safe, supportive, and empowering environments for all migrants and their families
- Review and share best practices on health promotion and preventive measures for migrant workers
- Promote partnerships and collaboration among local, national, and international agencies that deal with migrants

## Discussion

There are numerous reasons universities should be actively engaged in local, regional and global migrations activities. Not only do universities have a mandate to address complex societal challenges through education, research and service; but in the case of international migration, universities are a significant driver, and benefactor, of cross-border migration for education and employment. The Manila Declaration on Migration and Health provides an outline for universities in the Pacific Rim and beyond to address international migration. First, the Manila Declaration recognizes the role of universities as a key host of many migrants and their families. As such, universities must ensure that their campuses are safe, supportive, and empowering environments for all migrant students, employees, and their families. This should include the provision of health services, legal support, and educational programs. Model policies and best practices developed at universities for migrants can and should be shared with other employers.

The Manila Declaration also focuses on the three traditional activities of universities: education, research, and service. In terms of education, the Declaration recognizes the need for universities to incorporate learning about migration throughout their curricula to sensitize their learning community about the complex challenges faced by migrants. Recommendations include developing new courses on the phenomenon of migration, implementing mandatory training programs for students and/or students and staff to prevent discrimination and promote tolerance and inclusivity, creating staff positions or administrative offices that work with migrants, and a commitment to ensuring diversity in terms of the student and staff populations. Moreover, universities should aim to provide educational and training opportunities to migrants and their families in order to reduce socioeconomic disparities in immigrant communities. This can include providing scholarships to students or instituting employee recruitment targets for migrants. Finally, universities should also capitalize on their interdisciplinary research expertise to generate data on migrant experiences and ensure that this research is communicated to policymakers.

We should not underestimate the challenges of working across traditional boundaries and disciplines to address longstanding challenges on a global scale. Nor should we overestimate the value of research without action to address these challenges. Universities’ core service mandate should include building public awareness and sensitivity to migrants’ challenges and needs and developing policy frameworks appropriate to their diverse contexts that guide, promote, and reinforce commitment to migrant rights and health. The institutional values and power of universities allow for an influential platform for mobilization and change. The production of knowledge and evidence resulting from research will enhance understanding and assist in the development of improved solutions for migrants.

## Conclusion: our commitment to a concerted effort

APRU’s collaboration across different universities seeks to serve as a model for others regarding the global approaches necessary to address the needs and challenges of vulnerable populations. The commitments outlined in the Manila Declaration are aimed to break the cycle of health and human rights violations experienced by migrants around the world. It is imperative that universities recognize their role in global migration goes beyond knowledge production and dissemination. Universities help drive migration and are host to a significant portion of migrant students, employees, and their families. They benefit greatly from international migration and consequently should put in place model policies, procedures, and programs that support migrants’ health and well-being as a precursor to their significant social and economic contributions [7]. Universities have the responsibility to disseminate this information, and work as advocates to collaborate with communities, organizations, health professionals, and policymakers that share the mission to protect and promote migrant health. Ideally, APRU’s focus on this issue and the resulting Manila Declaration will result in other networks and institutions assessing their current practices and adopting similar measures to facilitate the inclusion of migrants in society.

## Data Availability

Not applicable.
